# Study on Flow Velocity Distribution in Open Channel With Flexible Vegetation

**DOI:** 10.3389/fpls.2021.753613

**Published:** 2021-11-22

**Authors:** Shiyu Wang, Yi Zhou, Tongshu Li, Song Li, Mingwu Zhang, Yu Han

**Affiliations:** ^1^College of Water Resources and Civil Engineering, China Agricultural University, Beijing, China; ^2^Yellow River Institute of Hydraulic Research, Yellow River Conservancy Commission, Zhengzhou, China

**Keywords:** flexible vegetation, velocity distribution, open channel, bursting phenomenon, mixing length

## Abstract

Ecological management of river channels is a hot topic for current sustainable development and flow measurement of ecological river is an important part. In this article, a flow velocity distribution model of the channel containing flexible vegetation is constructed from the vegetation riverbed theory and the bursting phenomenon to reveal the microscopic mechanism of the flow velocity distribution in the upper layer of vegetation. In the vegetation riverbed law, the effect of flexible vegetation is evaluated by the mixed length formula. The bursting phenomenon law considers the influence of the channel sidewalls on the flow and a two-dimensional velocity model is established by introducing the concept of average turbulence structure. The mechanism of the downward shift of the maximum flow velocity point on the channel sidewall is explained. The verification of the calculated velocity profiles is carried out based on data obtained in laboratory experiments. The results show that the combination of the two models can well describe the velocity distribution of the whole channel. At the end, the phenomenon of flow velocity zoning in open channel is discussed, which provides a solution for flow measurement in ecological channel.

## Introduction

Sustainable development is an important issue in the world. The presence of vegetation in river systems contributes to the sustainability of rivers and enhances the self-cleaning capacity of water (Lozanovska et al., 2020). However, the existence of vegetation will change the channel resistance and raise the water level (Rivaes et al., 2017). Ecological discharge is an important parameter in the ecological channel. The existence of vegetation causes some interference on the flow field and flow monitoring. Therefore, it is necessary to study the velocity distribution of the channel with flexible vegetation.

The effect of vegetation distribution on mean flow velocity and turbulence characteristics in a channel is generally studied by model experiments. Järvelä (2002) used natural aquatic grass, sedge, and willows as test material to study the influence of plants on flow velocity distribution by introducing plant parameters that characterize the flexibility and morphology of plants in flow. Bennett et al. (2002) analyzed the influence of vegetation belts arranged at intervals between two sides of water channel on flow characteristics by model test. It was found that vegetation belts carried flow to the other side and the intensity of flow were related to the density of vegetation. Wilson et al. (2005) developed a three-dimensional numerical model and compared it with a standard turbulent flow model to analyze the effect of willows on flow, pointing out that determining the degree of plant bending in flow is important for the study of the interaction mechanisms between vegetation and flow. Bouma et al. (2005) analyzed the influence of vegetation belt with specific shape on flow rate and turbulence and proposed that the distribution of flow rate is closely related to the spacing of vegetation belt. Neumeier (2005) measured the flow velocity and turbulence characteristics of the wake after it crossed the marsh vegetation and inferred that the larger vertical flow velocity and higher turbulence in front of the vegetation could reduce sediment deposition. Helmiö (2002) developed a one-dimensional model to solve the problem of composite channels with vegetation, in which the effect of flow distribution caused by vegetation was considered and the model was validated by using data from the Rhine river. He pointing out that vegetation density and the width of the diffuse zone have a large effect on the transport capacity of the main channel and significantly reduce the flood flow capacity of the channel. Leu et al. (2008) established a two-dimensional model to study the velocity distribution of five different submerged plants in different water depths, pointing out that the submerged plants can reduce the velocity and can meet the requirements of flood control. Zong and Nepf (2009) carried out model tests on flow in semi-covered vegetated river and discussed the changes of flow velocity and turbulence characteristics in vegetation and non-vegetation areas and found that flow can be divided into adjustment areas and fully developed areas. Siniscalchi et al. (2012) measured the flow characteristics of submerged flexible vegetation by flow channel experiment. It was found that the turbulence intensity corresponding to the front of vegetation and the top of vegetation increased and a negative Reynolds stress appeared in the vegetation layer, reflecting the influence of vegetation morphology on the flow velocity and turbulent flux. Wang et al. (2015) proposed a method to calculate the analytical solution of the vertical distribution of the mean flow velocity in the flow direction of an open channel containing flexible vegetation when a large bend occurs and derived a formula for the mean flow velocity of the linear flow resistance in the momentum equation. Okamoto et al. (2016) study how plant motions were coupled to strong oscillations in flow velocity associated with the “monami” phenomenon and its vertical extent in an open channel with flexible vegetation. Wang et al. (2016) simulated the water flow due to wind action on the water surface in a shallow lake through experiments. The effects of different densities of aquatic flexible vegetation on the hydrodynamic characteristics at different wind speeds were investigated. By analyzing the changes of vegetation drag (*C*_*d*_) and friction (*C*_*f*_) coefficient with flow and vegetation conditions under different test conditions, Pu et al. (2019) obtained a model of velocity distribution in open channel with flexible vegetation.

Although there are many studies on the hydrodynamic characteristics of flow with flexible vegetation, most of them are still limited to the corresponding hydrodynamic characteristics of specific vegetation. The characteristics of the flow field in the upper layer of vegetation have not been recognized after the flows through the vegetation and most of the current studies focused on the hydrodynamic characteristics of the flow in the center of the open channel. In fact, the hydrodynamic characteristics of the channel center and the open channel sidewalls are not the same. There are fewer studies on the phenomenon of downward shift of the maximum flow velocity point for the channel sidewall. The division of velocity zoning of channel section is not clear. The research results are not universal, so the research needs to be further strengthened.

This article studies on the velocity distribution law of ecological river with vegetation. Based on the vegetation riverbed theory and bursting phenomenon, the velocity distribution law of the whole section of the flow is established. Combined with the physical model test of ecological channel, the correctness of velocity distribution law is verified. Meanwhile, the flow velocity zoning phenomenon of the ecological open channel is discussed with the test results. It provides a solution for the flow measurement of ecological river.

## Modeling the Velocity Distribution in Channel With Flexible Vegetation

### Velocity Distribution Based on the Vegetation Riverbed Theory

Righetti and Armanini (2002) proposed that the vegetation layer can be regarded as a large-scale roughness and it is considered as a part of the riverbed. It is called the vegetation riverbed theory and the non-vegetation layer flows on this vegetation riverbed (Huai et al., 2009).

In this article, we focus on the velocity distribution of the non-vegetation layer and the initial velocity of the non-vegetation layer is the velocity of the top layer of vegetation. Therefore, it is necessary to know the velocity of the vegetation top layer. For the vegetation layer, the corrected gravity term of the water volume occupied by the flexible vegetation can be ignored. For a given water volume, plant-induced resistance per fluid mass can be described as (Kubrak et al., 2008):


(1) $$\text{F}=\frac{1}{2}\rho {\text{C}}_{d}{\text{mAu}}_{\mathrm{ud}}^{2}$$

Where, *ρ* is the density of water, *m* is the number of grass per unit area, *A* is the upstream area of vegetation, *u*_*ud*_ is the flow velocity at the top of the vegetation, and *C*_*d*_ is the drag force coefficient of the vegetation. For the drag coefficient of vegetation, the variation in vegetation layer from 1 to 1.5 was estimated from Kubrak et al. (2008) and Yang and Choi (2009) test data. It is found that Klopstra et al. (1997) proposed that it can be better applied to cylindrical vegetation. Therefore, this experiment is adopted *C*_*d*_ = 1.4.

The shear stress of uniform flow in open channel can be expressed as:


(2) $$\tau =\rho {\text{u}}_{*}^{2}$$

Where, *τ* is the shear stress, u_*_ is the friction velocity and it can be expressed as (Tracy and Lester, 1961):


(3) $${\text{u}}_{*}=\sqrt{\text{gHS}}$$

Where, *H* is the depth of water and *S* is the slope of the channel. Since the vegetation layer is not a uniform flow, it is necessary to multiply a correction factor before Eq. (2), and combined with Eq. (1) can be obtained that:


(4) $$\alpha \rho {\text{u}}_{*}^{2}=\frac{1}{2}\rho {\text{C}}_{d}{\text{mAu}}_{\mathrm{ud}}^{2}$$

Where, *α* is the correction coefficient and different correction coefficients can be got for different flexible vegetation. In this experiment, we can take $\alpha =7\times \frac{h_{v}}{H}$, where *h*_*v*_ is the height of deflected vegetation. Eq. (4) can be simplified to get the expression of the top flow velocity of vegetation.


(5) $${\text{u}}_{\mathrm{ud}}=\alpha \sqrt{\frac{2{\text{u}}_{*}^{2}}{{\text{C}}_{d}\text{mA}}}$$

For the non-vegetated layer, the equation of the flow under constant uniform flow conditions according to the force balance principle is:


(6) $$\frac{\partial \tau }{\partial \text{y}}+\rho \text{gS}=0$$

Neglecting the viscous stress and integrating Eq. (6), the distribution relation of the tangential stress is obtained as:


(7) τ=ρgS(H-y)

Prandtl (1925) assumes that the distance displaced by the momentum of the fluid mass before it is changed by the new environment is *l*. This is Prandtl’s mixing-length theory. According to Prandtl’s mixing-length theory, there are:


(8) $$\frac{\tau }{\rho }={\text{l}}^{2}{\left(\frac{\partial \text{u}}{\partial \text{y}}\right)}^{2}$$

According to the vegetation riverbed theory, the original channel is partly occupied by vegetation and the flow seems to be “compressed” and the expression of the mixing length should reflect this “compression” (Huai et al., 2009). A new mixed length expression is assumed as follows:


(9) $$\text{l}=\left(\frac{\text{H}-{\text{h}}_{v}}{\text{H}}\right)\kappa \text{y}\sqrt{\frac{\tau }{\tau_{\max}}}$$

According to Eq. (7), we get:


(10) $$\tau_{\max}=\rho \text{gSH}$$

Combining Eqs. (8–10), it is obtained that:


(11) $$\text{du}=\frac{1}{\kappa }\frac{\text{H}}{\text{H}-{\text{h}}_{v}}\sqrt{\text{gSH}}\frac{1}{\text{y}}\text{dy}$$

The integral is organized to obtain:


(12) $$\frac{\text{u}}{{\text{u}}_{*}}=\frac{1}{\kappa }\frac{\text{H}}{\left(\text{H}-{\text{h}}_{v}\right)}\ln\left(\frac{\text{y}}{{\text{h}}_{v}}\right)+\frac{{\text{u}}_{\mathrm{ud}}}{{\text{u}}_{*}}$$

Equation (12) is the theoretical model of flow distribution in open channels containing vegetation based on the vegetation riverbed theory. This velocity equation is named as the vegetation riverbed law (VRL).

### Velocity Distribution Based on the Bursting Phenomenon

As also mentioned earlier it can be proposed that the vegetation layer can be considered as a kind of rough object with a large scale, we can assume that the flexible vegetation is a special kind of boundary layer where turbulence can lead to the emergence of coherent structures. Nezu et al. (1993) introduced the concept of bursting phenomenon. The bursting phenomenon refers to the phenomenon that the boundary layer will suddenly rupture locally at a certain location in space when there is a strong interaction between the inner and outer regions of the boundary layer. In other words, the upthrow phenomenon of low-speed fluid and the down sweep phenomenon of high-speed fluid in wall turbulence are called bursting phenomenon. In fact, vortices originating from the wall region can be observed in the turbulent core and even at the free surface. This vortex is also intermittent and it is randomly distributed in size and direction (Yang, 2010).

For turbulent flows, the expressions for the mean Reynolds Navier–Stokes equations and the continuum equations are as follows:


(13) $$\begin{array}{c} u\left(\frac{\partial u}{\partial x}\right)+v\left(\frac{\partial u}{\partial y}\right)+w\left(\frac{\partial u}{\partial z}\right)=gS+\frac{\partial }{\partial x}\left(\bar{-u^{\prime }u^{\prime }}\right)\\ +\frac{\partial }{\partial y}\left(\bar{-u^{\prime }v^{\prime }}\right)+\frac{\partial }{\partial z}\left(\bar{-u^{\prime }w^{\prime }}\right)+\mu \left(\frac{\partial^{2}u}{\partial x^{2}}+\frac{\partial^{2}u}{\partial y^{2}}+\frac{\partial^{2}u}{\partial z^{2}}\right) \end{array}$$

(14) $$\frac{\partial \text{u}}{\partial \text{x}}+\frac{\partial \text{v}}{\partial \text{y}}+\frac{\partial \text{w}}{\partial \text{z}}=0$$

Where, *u*, *v*, and *w* represent the flow velocity along the flow direction *x*, *y*, and *z*. ν is the kinematic viscosity, $\bar{-u^{\prime }u^{\prime }}$, $\bar{-u^{\prime }v^{\prime }}$, $\bar{-u^{\prime }w^{\prime }}$ is the turbulent shear stress. For uniform flow, the variation in *x* direction can be disregarded and Eq. (13) can be written as:


(15) $$\begin{array}{c} \text{v}\left(\frac{\partial \text{u}}{\partial \text{y}}\right)+\text{w}\left(\frac{\partial \text{u}}{\partial \text{z}}\right)=\text{gS}+\frac{\partial }{\partial \text{y}}\left(\bar{--{\text{u}}^{\prime }{\text{v}}^{\prime }}\right)+\frac{\partial }{\partial \text{z}}\left(\bar{--{\text{u}}^{\prime }{\text{w}}^{\prime }}\right)\\ +\mu \left(\frac{\partial^{2}\text{u}}{\partial {\text{y}}^{2}}+\frac{\partial^{2}\text{u}}{\partial {\text{z}}^{2}}\right) \end{array}$$

In 1883, Reynolds decomposed the instantaneous velocities into two contributions—mean velocities and velocity fluctuations. Equation (15) can be further written as:


(16) $$\frac{\partial \left(uv-\frac{\tau_{xy}}{\rho }\right)}{\partial y}+\frac{\partial \left(uv-\frac{\tau_{xz}}{\rho }\right)}{\partial z}=\text{gS}$$

Where, $\tau_{xy}=\eta \frac{\partial u}{\partial y}-\rho \bar{u^{\prime }v^{\prime }}$, $\tau_{xz}=\eta \frac{\partial u}{\partial z}-\rho \bar{u^{\prime }w^{\prime }}$, and *η* is the kinetic viscosity.

In the two-dimensional flow, the first term on the left side of the equation in Eq. (16) should dominate and the second term has little effect and can be neglected. Eq. (16) can be simplified and integrated to obtain:


(17) $$\mu \frac{\text{du}}{\text{dy}}-\text{uv}-\bar{{\text{u}}^{\prime }{\text{v}}^{\prime }}=-\text{gSy}+\text{C}$$

Substituting the boundary conditions, Eq. (17) can be obtained as follows:


(18) $$\frac{{\text{du}}^{+}}{{\text{dy}}^{+}}-{\text{u}}^{+}{\text{v}}^{+}-\frac{\bar{{\text{u}}^{\prime }{\text{v}}^{\prime }}}{{\text{u}}_{*}^{2}}=1-\frac{\text{y}}{\text{H}}$$

Where, *u*^+^ = *u*/u_*_, *y*^+^ = yu_*_/*μ*, and *v*^+^ = *v*/u_*_.

A method for calculating the average turbulence structure is proposed here, where the probability of bursting phenomena *r* is first determined based on the direction of the vertical flow velocity. If the instantaneous velocity is greater than the average velocity, the subscript is recorded as *u*; otherwise, it is recorded as *d*. Where *r* is defined as:


(19) $${\text{r}}_{u}=\frac{\sum_{0}^{T_{u}}\Delta {\text{t}}_{u}}{\text{T}}$$

(20) $${\text{r}}_{d}=\frac{\sum_{0}^{T_{d}}\Delta {\text{t}}_{d}}{\text{T}}$$

Equation (19, 20) can be rewritten as:


(21) $${\text{r}}_{u}{\text{v}}_{u}+{\text{r}}_{d}{\text{v}}_{d}=0$$

The two-dimensional velocity affected by vegetation bursting can be defined as follows:


(22) $$u_{u}=\frac{1}{T_{u}}\int_{0}^{T_{u}}\tilde{u_{u}}\mathrm{dt}$$

(23) $$u_{d}=\frac{1}{T_{d}}\int_{0}^{T_{d}}\tilde{u_{d}}\mathrm{dt}$$

(24) $$v_{u}=\frac{1}{T_{u}}\int_{0}^{T_{u}}\tilde{v_{u}}\mathrm{dt}$$

(25) $$v_{d}=\frac{1}{T_{d}}\int_{0}^{T_{d}}\tilde{v_{d}}\mathrm{dt}$$

Where, $\tilde{u}$ is the instantaneous velocity. In Eq. (18), the formula of Reynolds shear force can be obtained by introducing the bursting phenomenon.


(26) $$\frac{{\text{du}}_{u}^{+}}{{\text{dy}}^{+}}-\frac{\bar{{\text{u}}_{u}^{\prime }{\text{v}}_{u}^{\prime }}}{{\text{u}}_{*u}^{2}}=\left(1-\frac{\text{y}}{\text{H}}\right)+{\text{u}}_{u}^{+}{\text{v}}_{u}^{+}$$

(27) $$\frac{{\text{du}}_{d}^{+}}{{\text{dy}}^{+}}-\frac{\bar{{\text{u}}_{d}^{\prime }{\text{v}}_{d}^{\prime }}}{{\text{u}}_{*d}^{2}}=\left(1-\frac{\text{y}}{\text{H}}\right)+{\text{u}}_{d}^{+}{\text{v}}_{d}^{+}$$

Where, $u_{u}^{\prime }=u-u_{u}$, $u_{d}^{\prime }=\tilde{u}-u_{d}$, $v_{u}^{\prime }=\tilde{v}-v_{u}$, and $v_{d}^{\prime }=\tilde{v}-v_{d}$ are the average turbulent structure; here $u_{u}^{+}=u_{u}/u_{*u}$ and $u_{d}^{+}=u_{d}/u_{*d}$, u_*u_ and u_*d_ are the local frictional flow velocity in the case of upward turbulence and downward turbulence of the flow.

According to Yalin (1977), the viscosity of vortices in water can be expressed as follows:


(28) $$\frac{\nu_{T}}{{\text{u}}_{*}}=\kappa \xi \left(1-\xi \right)$$

Where, *ν*_*t*_ is the vortex viscosity, which can be approximated to be equal to *μ*, *ξ* = *y*/*H*, and *κ* is the Carmen constant. At *y* = 0, due to the non-slip boundary condition (*u*_*u*_ = 0 and *v*_*u*_ = 0), the influence of additional momentum flux *u*_*u*_*v*_*u*_ on the velocity profile is negligible in the near-wall region. Substituting Eq. (28) into Eq. (26) and the non-slip boundary condition and simplify it:


(29) $$\frac{{\text{du}}_{u}^{+}}{\text{d}\xi }=\frac{\left(1-\xi \right)}{\kappa \xi \left(1-\xi \right)}+\frac{{\text{u}}_{u}^{+}{\text{v}}_{u}^{+}}{\kappa \xi \left(1-\xi \right)}$$

Similarly, it can be obtained:


(30) $$\frac{{\text{du}}_{d}^{+}}{\text{d}\xi }=\frac{\left(1-\xi \right)}{\kappa \xi \left(1-\xi \right)}+\frac{{\text{u}}_{d}^{+}{\text{v}}_{d}^{+}}{\kappa \xi \left(1-\xi \right)}$$

Equation (21) can be rewritten as:


(31) $${\text{v}}^{+}=r_{u}{\text{v}}_{u}^{+}{\text{u}}_{*u}+r_{d}{\text{v}}_{d}^{+}{\text{u}}_{*d}=0$$

The vegetation influence is stochastic, so the average velocity gradient based on the probability of bursting phenomenon *r* can be expressed as:


(32) $${\text{u}}^{+}=\frac{\text{u}}{{\text{u}}_{*}}=\frac{{\text{r}}_{u}{\text{u}}_{u}+{\text{r}}_{d}{\text{u}}_{d}}{{\text{u}}_{*}}={\text{r}}_{u}{\text{u}}_{u}^{+}\frac{{\text{u}}_{*u}}{{\text{u}}_{*}}+{\text{r}}_{d}{\text{u}}_{d}^{+}\frac{{\text{u}}_{*d}}{{\text{u}}_{*}}$$

So,


(33) $$\frac{{\text{du}}^{+}}{\text{d}\xi }={\text{r}}_{u}\frac{{\text{u}}_{*u}}{{\text{u}}_{*}}\frac{{\text{du}}_{u}^{+}}{\text{d}\xi }+{\text{r}}_{d}\frac{{\text{u}}_{*d}}{{\text{u}}_{*}}\frac{{\text{du}}_{d}^{+}}{\text{d}\xi }$$

By associating Eq. (33) with Eq. (29) and Eq. (30), the following results can be obtained:


(34) $$\frac{{\text{du}}^{+}}{\text{d}\xi }=\frac{1}{\kappa \xi }+\frac{A}{\kappa \xi \left(1-\xi \right)}$$

Where,


(35) $$\text{A}={\text{r}}_{u}\frac{{\text{u}}_{*u}}{{\text{u}}_{*}}\left({\text{u}}_{u}^{+}{\text{v}}_{u}^{+}-{\text{u}}_{d}^{+}{\text{v}}_{u}^{+}\right)$$

Integrating Eq. (35) and simplifying it gives:


(36) $$\frac{\text{u}}{{\text{u}}_{*}}=\frac{1}{\kappa }\ln\frac{\text{y}}{{\text{h}}_{v}}+\frac{\text{A}}{\kappa }\ln\frac{\text{y}}{{\text{h}}_{v}}-\frac{\text{A}}{\kappa }\ln\left(\frac{\text{H}-{\text{h}}_{v}}{\text{H}-\text{y}}\right)+\frac{{\text{u}}_{\mathrm{ud}}}{{\text{u}}_{*}}$$

Equation (36) is the theoretical model of flow velocity distribution in open channels with flexible vegetation based on the bursting phenomenon. This velocity equation is named as the bursting phenomenon law (BPL).

## Experiment

**FIGURE 1:**
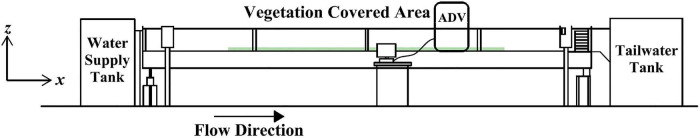
Experimental platform for physical model of ecological channel.

The model experiment was carried out on the open channel device in the hydraulic public test hall of the China Agricultural University. The rectangular channel is 6 m long, 0.8 m wide, 0.6 m high, and the slope is 5‰ (Figure 1). In order to ensure that the flow at the water inlet is as uniform as possible, the water inlet tank is aligned with the center line of the channel and a turbulent honeycomb is arranged at the water inlet of the tank. This experiment controls the water depth in the channel by controlling the valve system on the return water pipeline, so that the flow in the whole channel section can easily reach the uniform flow state in the open channel.

**FIGURE 2:**
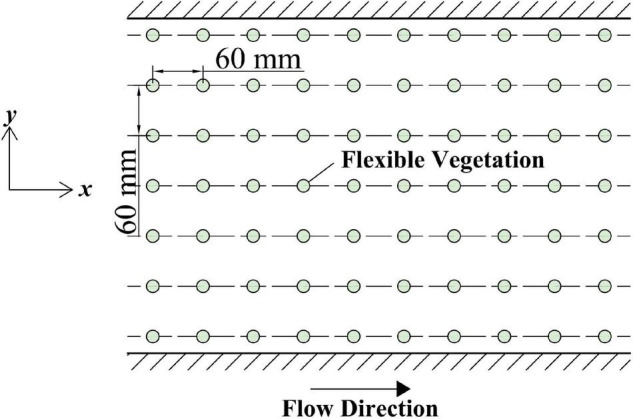
Vegetation arrangement diagram.

In this experiment, the flexible vegetation was simulated by plastic water plants; every plant was 0.06 m high, 0.03 m in diameter, and the total length of the vegetation section was 4 m. The vegetation is glued to the prefabricated perforated plastic plate, which is laid at the bottom of the sink. The vegetation strip was a single row of vegetation and equidistant (Figure 2); then, the position of the vegetation above the vegetation was measured with the plant as the reference.

The three-dimensional ultrasonic acoustic Doppler velocimetry (ADV) is used in this experiment to measure the velocity and ADV can measure the three-dimensional flow field information. The velocity range of ADV is from 0.001 to 4.5 m/s, the resolution can reach 0.0001 m/s, and the relative error is <1% of the measured velocity. To minimize the influence of ADV noise, the sample size of each measurement point in this experiment is about 2,000 times and the experiment time is 200 s. The downstream position is selected to ensure the stability of vegetation flow conditions.

**FIGURE 3:**
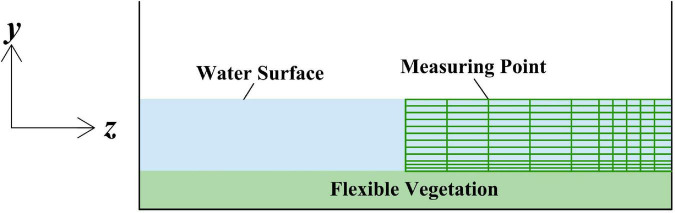
Open channel mesh measuring.

The section of rectangular channel is symmetrical and the velocity distribution of the section is also symmetrical, so only half of the test data with the vertical line as the boundary line in the open channel need to be measured. Each measuring point is a predivided grid node. It is proposed to arrange one measuring line from the center vertical line to the right at every 0.04 m and one measuring point at every 0.01 m and to encrypt appropriately when close to vegetation (Figure 3), so as to ensure that the measured data can accurately reflect the actual flow field. Four flow conditions are set in the test. The specific test conditions are shown in Table 1.

**TABLE 1:** Experiment conditions.

Case	Stem spacing (m)	Vegetation density (flexible stems/m^2^)	Flow *Q* (m^3^/h)	Depth *H* (m)	Height of deflected stem *h*_*v*_ (m)
1	0.06	272	90	0.159	0.0551
2	0.06	272	100	0.170	0.0541
3	0.06	272	110	0.181	0.0533
4	0.06	272	120	0.191	0.0522

## Results

**FIGURE 4:**
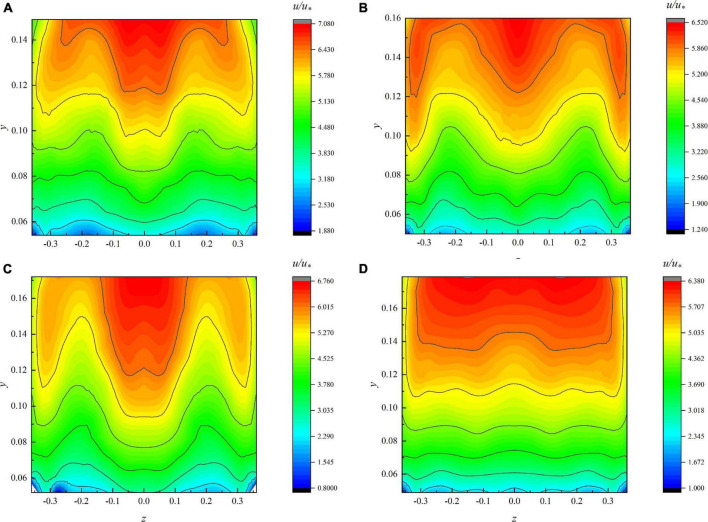
Flow field contour. **(A)** 90 m^3^/h, **(B)** 100 m^3^/h, **(C)** 110 m^3^/h, **(D)** 120 m^3^/h.

The experiment is completed based on the experimental platform in section “Experiment” and the measured data under different flow conditions are drawn into the contour (Figure 4). It can be seen from Figure 4 that the shape of the velocity curve is similar under each condition. The velocity distribution near the vertical line in the open channel generally decreases more evenly from the water surface to the vegetation as shown in Figure 4A; in other words, the maximum velocity point appears near the water surface. In the region near the sidewall, for the same *z*-coordinate of the velocimetric plumb line, it can be found that the velocity near the water surface is not the maximum and the maximum velocity point moves downward. There is also an appeal phenomenon in Figures 4B–D. It shows that it is not well considered to use a velocity formula to describe the velocity distribution of the whole section (Yang et al., 2004). Based on the antecedent analysis of contour, we use the experimental data to verify the theoretical formula derived in section “Modeling the Velocity Distribution in Channel With Flexible Vegetation.”

### Velocity Distribution in Channel Centerline

**FIGURE 5:**
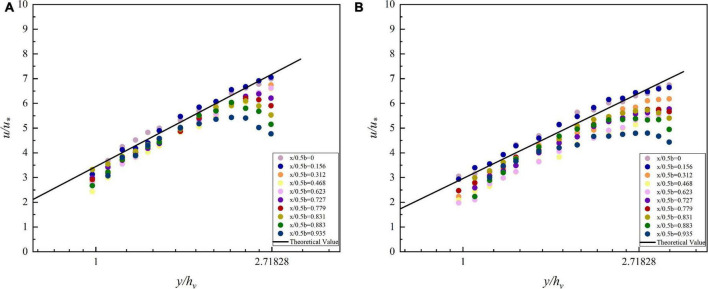
Flow velocity distribution of different velocity measurement plumb lines in vegetated open channel cross sections. **(A)** 90 m^3^/h, **(B)** 110 m^3^/h.

To explain the above phenomenon, the velocity distribution near the channel center line is discussed first. The velocity and depth are dimensionless, since they vary in size in each data series. By substituting the corresponding parameters into the VRL, the theoretical values of the flow velocity can be calculated. The theoretical values are plotted as straight lines, while the scatter points of the measured values are plotted in Figure 5. The scattered points are uniformly distributed around the straight line, indicating that the vegetated streambed flow distribution model is accurate in predicting the vertical distribution of flow near the center of the open channel containing flexible vegetation.

**FIGURE 6:**
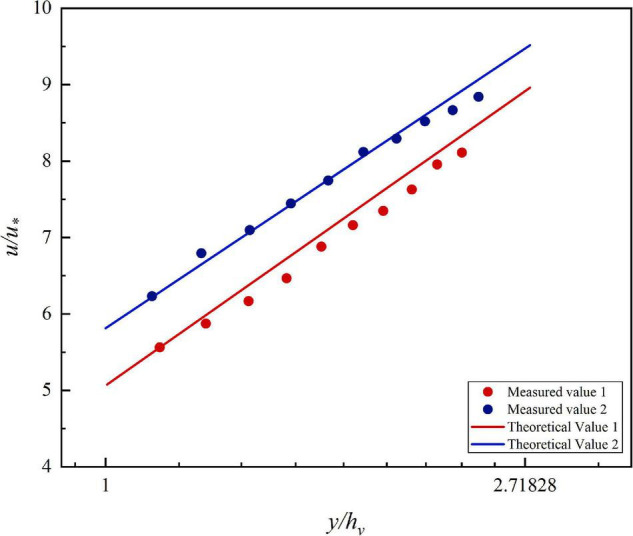
Comparison of the velocity distribution of open channel with vegetation measured by Yang and the velocity distribution model of vegetation layer theory.

To prove the generalizability of the VRL, the velocity distribution measured by Yang and Choi (2009) is redrawn in Figure 6. Yang measured the velocity distribution in the center of the channel under the condition of uniform flow. Two different typical flow conditions (27 m^3^/h and 37.8 m^3^/h) are selected. The blue and red scattered points in Figure 6 represent the dimensionless flow velocity under the two conditions and the red and blue lines represent the theoretical values calculated by applying the VRL under the corresponding conditions. Figure 6 clearly shows that the theoretical values are in good agreement with the measured data, indicating that the VRL is generally applicable in the channel near the center of the channel is generally applicable.

However, in Figure 5, it is noticed that the velocity near the water surface deviates from the straight line in the near sidewall area. The velocity near the measured point changes from an upward trend to a downward trend, which is consistent with that shown in the flow field contour. It means that the VRL cannot be applied in this region. Next, the velocity distribution in this region is discussed by using the formula of bursting phenomenon.

### Velocity Distribution of Channel Sidewall

This subsection focuses on the velocity distribution near the water surface.

In the experiment, it is found that the maximum point of velocity begins to move down from 0.1 m away from the sidewall and the VRL cannot well describe the velocity distribution in this area. It shows that the influence of vertical velocity should be considered. Therefore, the velocity distribution in this region should be calculated by the BPL.

**FIGURE 7:**
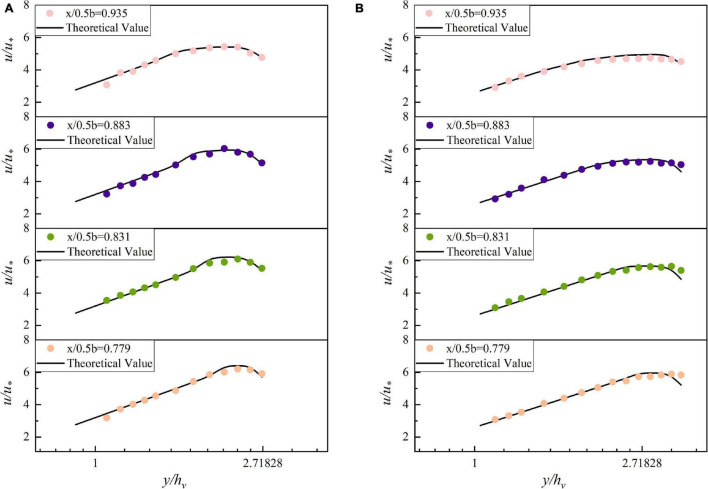
Comparison of flow velocity analysis in the near sidewall area. **(A)** 90 m^3^/h, **(B)** 120 m^3^/h.

The four-flow measurement vertical lines with the more obvious trend of decreasing flow velocity in the near sidewall area were selected for analysis under each operating condition. The theoretical value of velocity far from the water surface is calculated by the VRL and the BPL is used to calculate the area near the water surface. The theoretical values are plotted as black lines and the scattered points are the dimensionless velocities under each working condition, which are plotted in Figure 7. It is clear from Figure 7 that the trend of the scatter is consistent with the curve. The average error between the theoretical and experimental values under different working conditions is below 4%. Take Figure 7B as an example, where the minimum error between the theoretical and experimental values is 0.1% and the maximum error is 9.2%. It shows that the model is more accurate to predict the velocity distribution near the water surface.

After the above analysis, it is shown that the flow velocity distribution of the whole section can be well described by using the VRL and the BPL in different areas.

## Discussion

In fact, it is because the flexible vegetation is inverted with the flow, which is equivalent to forming a “vegetation riverbed” with flexible vegetation as the bottom wall, so the flow in most non-vegetated areas still conforms to the logarithmic law. The biggest difference between the logarithmic velocity distribution formula of vegetated channel and smooth channel is the difference of “riverbed,” which is also reflected in the VRL. In the VRL, it is obvious that the denominator of logarithmic term is the height of deflected vegetation (*h*_*v*_), while the denominator of logarithmic term of traditional velocity distribution is the boundary layer separation point (Nikuradse, 1930). It shows that the height of deflected vegetation (*h*_*v*_) is the characteristic length of open channel with vegetation. The characteristic length has also been mentioned in the study by Ghisalberti and Nepf (2002) and they believe that it can be expressed by using the momentum thickness. The momentum thickness needs to be obtained from the flow velocity calculations at the upper and lower boundaries of the mixed layer. In contrast, the calculation of the VRL is simpler.

Similarly, the VRL and the BPL have some similarities. The first term of the two equations is the same; it shows that the BPL also reflects the influence of flexible vegetation on flow. The difference appears in the following expressions. In the VRL, only one-dimensional mainstream velocity is used for calculation. The experimental results show that the flow near the center of the channel is closer to uniform flow and only the influence of the main flow direction can be considered. Most of the flow velocity equations proposed by researchers nowadays only consider the effect of flow velocity in the mainstream direction. For example, the flow velocity equation proposed by Nepf (2011) is partitioned for the vertical direction, but not for the cross-sectional flow velocity of the channel. However, there is a significant downward shift of the maximum velocity point in the region close to the sidewall. It is usually considered that the flow is influenced by the sidewall and produces a secondary flow. The manifestation of secondary flow is that there is a vortex near the water surface, which has a strong horizontal flow pointing to the center. This is the main reason for the location of the maximum flow velocity below the free surface (Nezu and Rodi, 1986). So, the vertical velocity should be included in the calculation. The BPL has a coefficient *A*, which includes the influence of mainstream velocity and vertical velocity.

**FIGURE 8:**
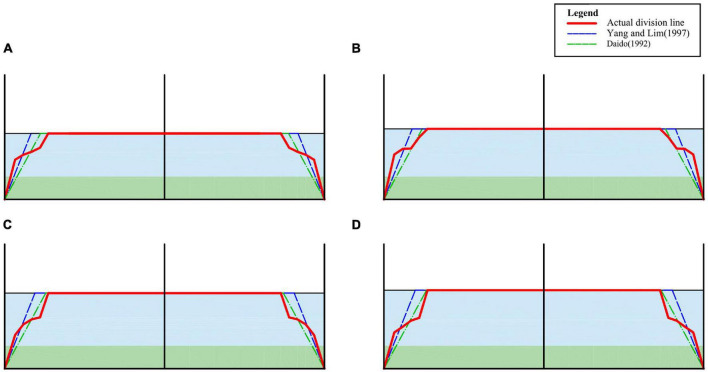
Velocity dividing line of open channel with flexible vegetation. **(A)** 90 m^3^/h, **(B)** 100 m^3^/h, **(C)** 110 m^3^/h, **(D)** 120 m^3^/h.

Through the above analysis, it can be found that the flow velocity of open channel has obvious zoning phenomenon in the cross section. Take the maximum velocity point on the velocity measuring vertical line as the dividing point, next connect the dividing points on each vertical line and draw the red dividing line in Figure 8. The region above the dividing line is the region affected by the sidewall and the velocity in this region can be calculated by the BPL. The velocity in the area below the dividing line can be calculated by the VRL. The form of the theoretical partition line proposed by Daido (1992) and Yang and Lim (1997), who considered that the form of the dividing line is related to the roughness ratio of the sidewall to the sidewall and is plotted in their proposed theoretical dividing line in the figure (blue and brown lines). Figure 8 shows that there is a gap between the theoretical dividing line and the actual dividing line clearly. The theoretical dividing line is a straight line near the sidewall, while the actual dividing line is a curve. Taking Figure 8A as an example, the dividing line in the near sidewall region has two particularly obvious inflection points, which is the same as that in Figures 8B–D. It shows that the split line is not a simple primary function form, but has a more complex function form, which will be discussed in depth in future studies.

## Conclusion

This article studies the flow velocity distribution law with flexible vegetation theoretically and verifies the vegetation riverbed theory and the bursting phenomenon theory based on specific experimental data. The similarities and differences between the VRL and the BPL are also discussed. Based on the section dividing theory, the velocity dividing line of open channel with flexible vegetation is discussed. The main conclusions can be summarized as follows:

(1) Based on the vegetation riverbed theory, the velocity distribution formula of open channel with vegetation near the channel center can be derived. The equation shows that the vegetation in the open channel can be regarded as a “vegetation riverbed” and the flow in the upper layer of vegetation flows over the riverbed. The experimental data also confirm this hypothesis.
(2) The equation of flow velocity distribution in maximum velocity point drop was derived by using the bursting phenomenon theory. The BPL shows that the flow in the near sidewall area will be affected by secondary flow and the influence of the vertical flow velocity of the water needs to be considered. There are some similarities between the VRL and the BPL. Compared with the functional form of the VRL, the BPL has one more wake function. The correctness of the BPL is proved by the test data.
(3) There is an obvious zoning phenomenon in the flow velocity of the cross section of the open channel with flexible vegetation. The open channel velocity dividing partition line was found by the maximum velocity point of different velocity measurement plumb lines. Compared with the smooth open channel dividing line, the partition line with flexible vegetation has a more complex functional form. It provides a theoretical basis for the flow measurement of ecological open channel.

## Data Availability Statement

The raw data supporting the conclusions of this article will be made available by the authors, without undue reservation.

## Author Contributions

SW contributed to the conceptualization, methodology, formal analysis, investigation, resources, data curation, writing—original draft, and visualization. YZ contributed to the investigation, resources, and data curation. TL contributed to the conceptualization, resources, writing—review and editing, and supervision. SL contributed to the investigation and resources. MZ contributed to the writing—review and editing. YH contributed to the conceptualization, validation, writing—review and editing, supervision, and funding acquisition. All authors contributed to the article and approved the submitted version.

## Conflict of Interest

The authors declare that the research was conducted in the absence of any commercial or financial relationships that could be construed as a potential conflict of interest.

## Publisher’s Note

All claims expressed in this article are solely those of the authors and do not necessarily represent those of their affiliated organizations, or those of the publisher, the editors and the reviewers. Any product that may be evaluated in this article, or claim that may be made by its manufacturer, is not guaranteed or endorsed by the publisher.
